# Does chronic jet lag increase risk of cancer?

**DOI:** 10.18632/aging.203596

**Published:** 2021-09-28

**Authors:** Suliman Khan, Mengzhou Xue, V. Wee Yong

**Affiliations:** 1 Department of Cerebrovascular Diseases, The Second Affiliated Hospital of Zhengzhou University, Zhengzhou, Henan, China; 2 Hotchkiss Brain Institute and Department of Clinical Neurosciences, University of Calgary, Calgary, Alberta, Canada

**Keywords:** circadian rhythm, glioma, immunity, microglia, transcriptome

Disrupted circadian rhythms negatively affect physiology and elevate the risk of immune and proliferative diseases [1]. The master timekeeper located in the suprachiasmatic nucleus of the hypothalamus is entrained by light to regulate peripheral clocks. Frequent trans-meridian flights or night-shift work challenges the circadian clock, and modeling this in mice through ‘chronic jet lag’ accelerates the growth of osteosarcoma [2]. We recently described that chronic jet lag in mice, induced by advancing the light-dark cycle over long periods, alters brain transcriptomes that have been linked to cancer-related pathways; our results suggest that chronic jet lag may increase susceptibility to cancer by altering molecular and metabolic processes [3]. In another study in mice, we determined that chronic jet lag changes the expression of genes related to glioma (a brain tumor) in several brain regions (nucleus accumbens, hippocampus, prefrontal cortex, hypothalamus and striatum) of wild-type and mutant (clock gene knockout) mice [4].

Collectively, the above results invite the hypothesis that prolonged disruption of circadian rhythms, such as by chronic jet lag, may lay the foundation for brain alterations that increase the susceptibility to brain tumors. Other reports support this hypothesis. Kettner et al. reported that chronic jet lag-mediated genome-wide gene deregulation increases the risk of hepatocellular carcinoma [5]. Hadadi et al. found that prolonged circadian disruption simulating chronic jet lag elevates the stemness and metastatic capacity of breast cancer cells in a mouse model of spontaneous mammary tumourigenesis; the chronic jet lag also generates an immune microenvironment that favors tumor growth [6].

Although the preclinical results that chronic jet lag alters brain transcriptomes linked to brain tumors [3,4] are interesting, the studies have shortcomings in that there was no demonstration that mice eventually developed brain tumors. Indeed, linking brain transcriptome changes to brain tumor formation is a difficult endeavor since other changes are necessary for the tumor to manifest. These disruptions could include further genetic changes, loss of mechanisms that reduce the capacity to eliminate a cell undergoing transformation, the contribution of age, and subjugation of the immune system. Nonetheless, there are results that shiftwork, which produces similar alterations in the circadian clock system, increases cancer risk in humans [7].

Despite the body’s timekeeper and peripheral clocks, almost every cell in the body has its own molecular clocks, which regulate the pace for cell decay, division, and growth. These clocks are likely to be altered in response to chronic jet lag, and thus can dysregulate the pace for cell growth to increase the risk of tumor generation. The functionality of immune cells is highly dependent on the circadian rhythm, and most immune cells contain their own molecular clocks. Thus, the normally protective immune system may becomes deficient or compromised in suppressing tumor development or progression in chronic jet lag. In the case of brain tumors, the circadian rhythm of microglia [8] may be similarly affected by chronic jet lag; the result could be the loss of the microglia capacity to suppress the pro-tumorigenic transcriptomic changes [3,4] resulting from chronic jet lag.

If chronic jet lag predisposes to cancer, how may this be prevented? One avenue is to reprogram the molecular clock to mitigate the associated risks. This would require uncovering the molecular mechanisms that links the altered circadian rhythm of chronic jet lag to cancer-related genes; strategies to counter the molecular mechanisms could then be conceived. Another approach is to determine the immune repertoire altered by chronic jet lag, so as to preserve the role of the immune system to curb tumorigenesis. Another line of investigation is to determine in several models whether chronic jet lag does indeed result in tumor initiation and progression. For now, one needs to be mindful of the possible link of prolonged circadian disruption through chronic jet lag and the altered brain transcriptomes of cancer-related pathways [3,4].

## References

[1] Aiello I, et al. Sci Adv. 2020; 6:eaaz4530. 10.1126/sciadv.aaz4530 33055171 PMC7556830

[2] Filipski E, et al. Cancer Res. 2004; 64:7879–85. 10.1158/0008-5472.CAN-04-0674 15520194

[3] Khan S, et al. Carcinogenesis. 2021; 42:864–73. 10.1093/carcin/bgab012 33608694

[4] Khan S, et al. Int J Biol Sci. 2019; 15:1816–34. 10.7150/ijbs.35520 31523185 PMC6743288

[5] Kettner NM, et al. Cancer Cell. 2016; 30:909–24. 10.1016/j.ccell.2016.10.007 27889186 PMC5695235

[6] Hadadi E, et al. Nat Commun. 2020; 11:3193. 10.1038/s41467-020-16890-6 32581213 PMC7314789

[7] Hansen J Cancer Causes Control. 2006; 17:531–37. 10.1007/s10552-005-9006-5 16596307

[8] Madore C, et al. Immunity. 2020; 52:222–40. 10.1016/j.immuni.2019.12.003 31924476 PMC7234821

